# Chemoresistance and Cancer-Related Inflammation: Two Hallmarks of Cancer Connected by an Atypical Link, PKCζ

**DOI:** 10.3389/fonc.2013.00232

**Published:** 2013-09-12

**Authors:** Alessandro Rimessi, Simone Patergnani, Elli Ioannidi, Paolo Pinton

**Affiliations:** ^1^Section of Pathology, Oncology and Experimental Biology, Laboratory for Technologies of Advanced Therapies (LTTA), Department of Morphology, Surgery and Experimental Medicine, Interdisciplinary Center for the Study of Inflammation (ICSI), University of Ferrara, Ferrara, Italy

**Keywords:** atypical PKC, PKCζ, cancer, chemoresistance, inflammation, nucleus, apoptosis

## Abstract

Atypical protein kinase C isoforms are serine threonine kinases involved in various pathological conditions. In recent years, the PKCζ isoform has emerged as an important regulator of multiple cellular processes operating in cancer. In this review, we will focus on the PKCζ isoform as an oxidative-sensing kinase involved in cancer-related inflammation and chemoresistance. We will discuss its nuclear localization and its possible pivotal role in connecting inflammation with drug resistance.

## Introduction

The protein kinase C (PKC) family consists of serine/threonine kinases that can be grouped into three subfamilies based on their structure and activators (1–3). The proteins within the subfamilies differ in their primary structure, expression patterns, subcellular localization, *in vitro* activation, and responsiveness to extra-cellular signals, which suggests the existence of a complex molecular machinery that regulates the specific sorting of various isoforms.

Conventional PKCs are calcium dependent and are stimulated by the second messenger diacylglycerol. Novel PKCs are calcium independent but are also capable of being stimulated by diacylglycerol. However, atypical PKCs require neither calcium nor diacylglycerol for optimal activity (4) but, rather, are dependent on lipid components, such as phosphatidylinositols (PIs) (5), phosphatidic acid (6), arachidonic acid, and ceramide (7).

Inactive PKC is mainly present in the cytosol, whereas activated PKC is associated with the plasma membrane, nucleus, and other subcellular compartments (8–11). This differential localization or intracellular redistribution offers an important level of regulation of the kinase, favoring interactions with specific activators or substrates.

Researchers have demonstrated a role of oxidative stress in the activation and regulation of PKC. Oxidative stress is involved in the pathogenesis of various degenerative diseases, including cancer and inflammation (12–15). All of the isoforms of PKC contain regions in both the N-terminal regulatory domain and the C-terminal catalytic domain that are susceptible to redox modifications (2). The sensitivity of PKC regions to redox stress interferes with the physiological activity of PKCs, and thus, with their biological effects.

Aberrant regulation or altered expression of PKCs has been implicated in the development, progression, and maintenance of the neoplastic phenotype (16, 17). Thus, logical candidates for the mediation of the pathological transduction of redox stress in cancer and cancer-related events are the PKCs.

In recent years, the atypical PKCs, particularly the ζ isoform, have emerged as pivotal regulators of cellular processes operating in cancer. The aim of this review was to summarize the available knowledge on the PKCζ isoform in cancer and chemoresistance, thereby strengthening the link between PKCζ-dependent inflammation and chemosensitization.

## The Multidomain Structure of Atypical Protein Kinase C Isoforms

The atypical PKCs, which form a subgroup within the PKC family, consist of isoforms ι, λ, and ζ. PKCι and PKCλ are orthologs showing 98% overall amino acid sequence identity; hereafter, these proteins will be referred to as PKCι/λ.

**Figure 1:**
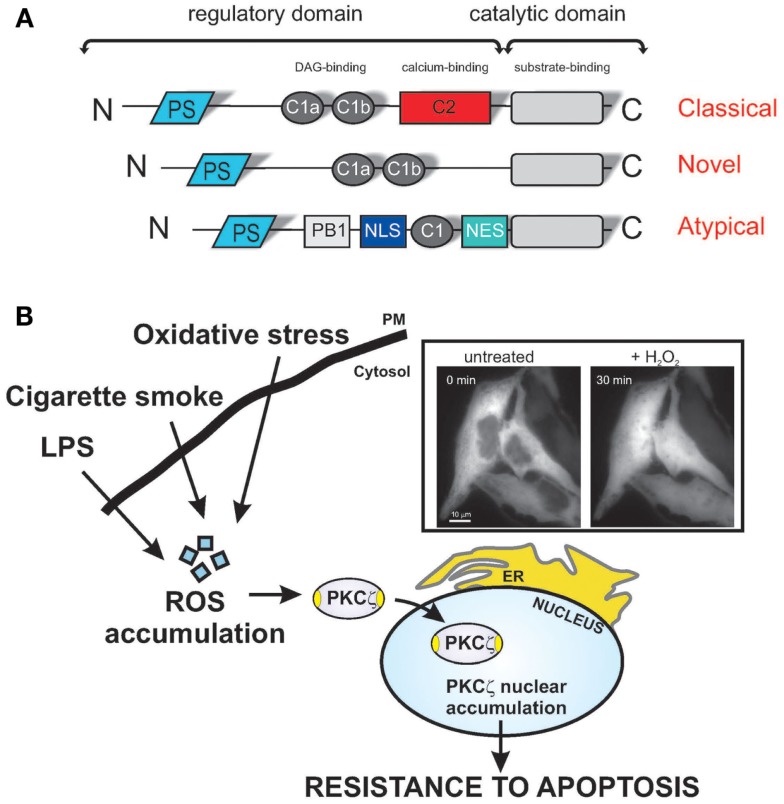
**Redox stress induces PKCζ nuclear translocation, protecting cells via various apoptotic stimuli**. **(A)** Schematic model of PKC structure. Representation of the different PKC subfamilies and their structural domains. The pseudo-substrate (PS) domain blocks the substrate-binding cavity of the kinase domain as an autoinhibitory mechanism. Conserved region 1 (C1) confers binding to diacylglycerol (DAG) and phospholipids, and C2 senses calcium. The PB1 and NLS/NES domains are specific for atypical PKCs and act as a protein-binding domain and nuclear import-system domain, respectively. **(B)** Shows representative images of chimeric PKCζGFP-expressing HeLa cells in the resting condition (untreated) and after a 30-min treatment with 1 mM H_2_O_2_. PKCζ localizes uniformly throughout the cytoplasm and is excluded from the nucleus, but upon oxidative stress challenge, the kinase translocates to the nucleus. Stress conditions, such the presence of as bacterial infection or cigarette smoke, favor the accumulation of intracellular ROS, and nuclear redistribution of the ζ isoform, conferring apoptotic resistance, and thus, chemoresistance.

Closer examination of protein sequence alignments between the PKC isoforms reveals sequence homology among the different members of this group. PKCζ and PKCι/λ consist of four functional domains, including a PB1 domain in the N-terminus, a pseudo-substrate (PS) domain, a C1 domain containing a single Cys-rich zinc finger motif, and a kinase domain at the C-terminus (Figure 1A) (10). The classical PKCs differ in their homologous domains (C2), which appears to be related to the Ca^2+^ sensitivity of the kinases. Both novel and atypical PKCs lack the C2 homologous domain and, thus, do not require Ca^2+^ for activation. In contrast, only the atypical PKCs additionally lack one-half of the C1 homologous domain (resulting in insensitivity to DAG) (Figure 1A).

The PB1 domain of PKCζ recognizes the OPCA motifs of PAR-6, ZIP/p62, and MEK5 (18). The PS domain blocks the substrate-binding cavity of the kinase domain as an autoinhibitory mechanism. The kinase domain of PKCζ and other members includes an ATP-binding region, an activation loop, a turn motif, and a hydrophobic motif. The ATP-binding region contains the Lys-281 residue, which is crucial for its kinase activity, while the activation loop and turn motif contain threonine residues (Thr-410 and Thr-560), which are phosphorylated upon activation. Finally, a nuclear localization signal (NLS) sequence is present in atypical PKCs that allows their rapid import into the nucleus via the formation of an NLS-importin complex (19), in addition to a short leucine-rich nuclear export signal (NES) sequence. NES-dependent nuclear export is inhibited by leptomycin B, which interferes with the binding of NES to CRM1/exportin 1 (20). The exposure of the NLS in PKCζ may be regulated by intra-molecular interactions between the N-terminal region and the catalytic domain of the kinase. However, these conserved domains confer specific localization and/or activation inputs for the isotypes.

## Atypical Protein Kinase C Isoforms in Cancer Biology

In the last decade, atypical PKCs have been implicated in carcinogenesis. Several studies have reported that PKCι/λ plays a key role in the promotion of carcinogenesis, both *in vitro* and *in vivo*. For example, PKCι/λ is implicated in Ras signaling, and in particular, PKCι/λ is required for oncogenic Ras-mediated colon carcinogenesis (21). In this study, transgenic mice expressing a constitutively active form of PKCι/λ displayed a significant number of pre-neoplastic lesions in the colonic epithelium (21). PKCι/λ is also important in cancer development and chemotherapy resistance in non-small cell lung cancers and human colon carcinomas, in which the kinase is highly expressed. In fact, the introduction of a dominant negative PKCι/λ mutant or inhibition of its expression was found to block oncogenic transformation and increase sensitivity to chemotherapeutic agents (22). In summary, PKCι/λ can be considered an important oncogenic molecule whose expression can be used as a prognostic marker for several human cancers (23). As reported above, PKCζ and PKCι/λ are members of the same group and exhibit 72% sequence homology at the amino acid level. However, PKCζ and PKCι/λ exhibit distinct functions, especially during cancer induction and maintenance, where PKCζ appears to play a controversial role in the neoplastic phenotype. PKCζ is not activated by diacylglycerol but is stimulated *in vitro* by the products of phosphatidylinositol 3-kinase (PI3-K), which strengthens its role in cellular proliferation. PKCζ interacts directly with Ras during mitogenic signaling. Ras has been demonstrated to interact *in vitro* with the regulatory domain of PKCζ and this association *in vivo* is triggered by platelet-derived growth factor (24).

A number of studies support the clinical relevance of PKCζ as a tumor suppressor, and a particular mutation in PKCζ has been found in human cancers (S514F) (25). The ability of PKCζ overexpression to restrain Ras-induced tumorigenesis is severely inhibited by the PKCζS514F mutation (26) It has been difficult to establish whether PKCζ is a pro- or anti-neoplastic protein, as a panel of human tumors was shown to exhibit contrasting protein expression levels of PKCζ (26, 27). Furthermore, an anti-apoptotic effect and, recently, a chemoresistant effect have been attributed to PKCζ (8).

In the following sections, we will attempt to elucidate the involvement of the ζ isoform in carcinogenesis and its putative role as a chemosensitizer.

## PKCζ and Its Role in Cancer Biology

As first described in 1999, increased expression of PKCζ is a characteristic of human prostate cancer (28). A subsequent study found that the induction of RNA interference against this kinase in PC3 prostate cancer cells reduced their malignant potential (29), confirming the critical role of PKCζ in promoting the malignant prostatic phenotype. Activation of the atypical kinase is not only necessary but also sufficient to deregulate growth control in mouse fibroblasts. Using a dominant kinase-defective mutant of PKCζ, the authors confirmed that the kinase is required for mitogenic activation in fibroblasts (30). Two studies have shown that PKCζ can promote the mobility of human MDA-MB-468 breast cancer cells and pancreatic cancer cells (31, 32). However, in these studies, the direct involvement of the ζ isoform in cancer progression was not well described because the authors only used PS peptide inhibitors and dominant negative mutants of atypical PKC.

Recently, an elegant study performed by Kim et al. showed how PKCζ induces the phosphorylation of c-Myc and the consequent inhibition of prostate tumorigenesis. Genetic inactivation of PKCζ in mice was reported to result in invasive prostate carcinomas *in vivo*, which was associated with increased cell growth, invasion, and metastasis; these findings revealed that the phosphorylation of c-Myc on Ser-373 by PKCζ is necessary and sufficient to repress c-Myc-activity (33). In 2013, a mechanism by which PKCζ regulates tumor metabolism was described. Here, a lack of PKCζ was found to be essential for reprograming the metabolism of tumor cells deprived of glucose through the utilization of glutamine. This work highlighted the major involvement of the serine biosynthetic cascade controlled by 3-phosphoglycerate dehydrogenase (PHGDH), which was recently shown to be significantly relevant in cancer (34). A recent study showed that overexpression of PKCζ inhibits human breast cancer (35), whereas the loss of this kinase promotes growth and colon tumor formation. To verify the hypothesis that PKCζ can promote transformed growth and colon tumor formation, a dominant negative, kinase-deficient PKCζ was overexpressed in CaCo_2_ human colon cancer cells, which stimulated soft agar growth (36). Moreover, the amount of PKCζ is significantly reduced in azoxymethane (AOM)-induced colon tumors in rats, and overexpression of PKCζ inhibits the growth of human MDA-MB-468 breast cancer cells (35).

Based on these reports, it is clear that PKCζ influences tumorigenesis through different molecular pathways that sustain proliferative signaling, allow evasion of growth suppressors, reprogram energetic metabolism, and activate invasion and metastasis. Two other pivotal hallmarks of cancer associated with PKCζ have been well examined, i.e., resistance to cell death and inflammation, both of which can be directly linked to the maintenance of the neoplastic phenotype.

We recently demonstrated that PKCζ induces resistance to apoptotic agents following its translocation into the nucleus as a result of oxidative stress (8). Supporting the importance of the role of the nuclear-PKCζ fraction in chemoresistance, we have shown that a recombinant nuclear-PKCζ inhibitor restores the apoptotic susceptibility of doxorubicin-resistant cells (Figure 1B). Indeed, we have provided direct evidence that doxorubicin-resistant cells present nuclear-PKCζ accumulation as a consequence of ROS accumulation (Figure 1B). The involvement of PKCζ through daunorubicin has also been described, where the activation of PKCζ triggers the Raf-1/MEK/ERK pathway (37) and inhibits the sphingomyelin-ceramide pathway, favoring daunorubicin-dependent chemoresistance (38).

This molecular pathway of chemoresistance is counteracted by Rituximab treatments, which inhibit the PKCζ/MAPK/mTOR pathway in follicular cell lymphoma (39). Treatment of lymphoma cell lines with Rituximab sensitizes the cells to the cytotoxic and apoptotic effects of therapeutic drugs, due partly to modification of the synthesis and secretion of anti-apoptotic cytokines implicated in drug resistance, including IL-6, IL-10, and TNFα, and to the inhibition of NF-κB activity (40, 41).

The “oncogenic behavior” of ROS has been substantiated by a growing body of evidence (42, 43). The ROS within cells act as secondary messengers in intracellular signaling cascades that induce and maintain the oncogenic phenotype of cancer cells, facilitating mutagenesis, tumor promotion, progression, and chemoresistance (44, 45). Oxidative stress induces PKC translocation, which is specific for different isoforms and different cell types. For example, in mouse embryonic fibroblasts (MEFs) and HeLa cells, oxidative stress triggers the translocation of the PKCα, β, δ, and ε isoforms from the cytosol to the plasma membrane (11). Under the same conditions, PKCζ translocates to the nucleus in MEFs (46) and HeLa cells (8). We previously described a functional role of nuclear PKCζ in the regulation of cell viability through the suppression of apoptotic cell death, thereby shifting the attention of researchers from cytosolic processes regulated by PKCζ, such as sphingomyelinase inactivation (38) or caspase 9 activation (47), to unknown nuclear events. These results support the link between the oncogenic behavior of ROS and the promotion of chemoresistance via nuclear-PKCζ translocation. Furthermore, this outcome confirms that nuclear PKCζ reduces the sensitivity of cancer cells to chemotherapeutic agents, thus supporting the usefulness of this kinase as a target for tumor cell chemosensitization.

Studies addressing lung cells and MonoMac6 cells exposed to cigarette smoke (a cancer inducer) or lipopolysaccharide (LPS, typically an inflammation inducer) showed that the levels of phosphorylated and total PKCζ increased in the nucleus, where phosphorylated PKCζ formed a complex with the pro-inflammatory transcription factor NF-κB (48).

## PKCζ and Inflammation

Over time, tumor cells can become resistant to anti-neoplastic drugs because molecular escape routes intervene to promote and maintain cancer integrity, thereby avoiding apoptosis or senescence pathways (49–52). A growing body of evidence indicates a role of the inflammatory tumor microenvironment in not only sustaining cancer development but also in cancer responsiveness and resistance to anticancer therapies (53).

**Figure 2:**
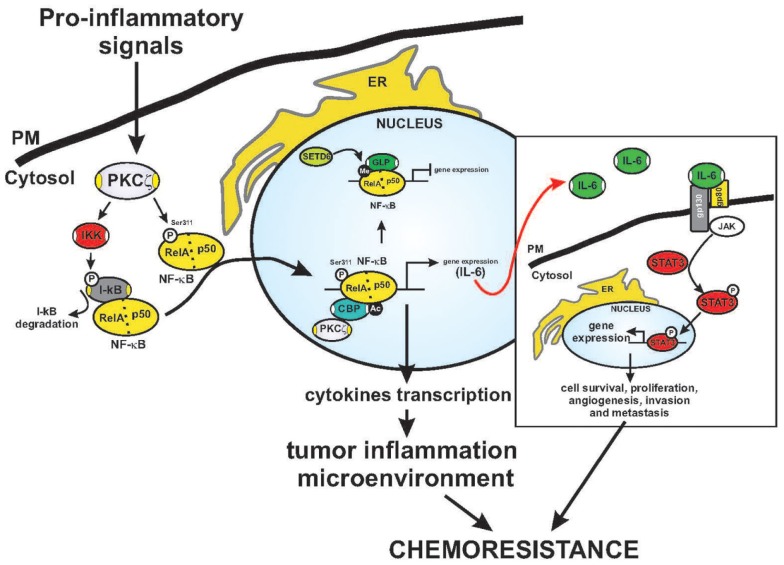
**Fine regulation of NF-κB activation by PKCζ. Schematic model of the regulation of PKCζ in the inflammatory response and chromatin remodeling**. Activated PKCζ may activate IKK kinase and trigger IκB degradation. This event precedes NF-κB activation and nuclear translocation, which makes NF-κB free to interact with elements in the promoters of inflammatory and survival genes. Indeed, the ζ isoform may directly interact with and phosphorylate the RelA subunit on Ser311 (P), leading to increased NF-κB transactivation. The Serine 311 residue is an important residue for recruiting the CBP coactivator complex. This event promotes acetylation (Ac) and the activation of cytokine transcription, that of including IL-6. Under basal conditions, RelA is methylated (Me) by SETD6, promoting the recruitment of GLP, which leads to repression of transcription. STAT3 is a key player in mediating inflammation-driven tumorigenesis, being constitutively activated by chronically high levels of the pro-inflammatory cytokine IL-6. In tumors, STAT3 is known to enhance cell survival and proliferation and to promote immune escape and angiogenesis, invasion, and metastasis. Once activated via tyrosine phosphorylation by receptor-associated JAK kinases, STAT concentrates in the nucleus and regulates the expression of target genes. The established inflammatory tumor microenvironment may contribute to the final outcome of the neoplastic process.

Several chemotherapeutic agents can activate the transcription factor NF-κB, thereby promoting chemoresistance through serine phosphorylation of the inhibitor (IKB kinase, IKK) of IκBα (54, 55). The functions of NF-κB, including transactivation, nuclear translocation, and DNA binding, are blocked by its cellular inhibitor, the IκBα protein. An essential component of the NF-κB pathway is the IKK complex, which phosphorylates IκBα and triggers its degradation, releasing NF-κB from its cytosolic state and promoting its translocation into the nucleus (56). PKCζ phosphorylates the IKKβ subunit *in vitro*, possibly through a direct interaction (Figure 2) (57). In HEK293 cells, PKCζ interacts with IKKβ at each catalytic domain in a TNFα stimulation-dependent manner, thereby activating IKK (57). In the lungs of PKCζ-deficient mice, TNFα-induced IKK activation is repressed (58). Indeed, PKCζ has been identified as a ceramide-activated protein kinase that is critical in stress-induced Jun N-terminal kinase activation and NF-κB translocation (59). In lung carcinogenesis, through its ability to activate NF-κB-dependent inflammation, PKCζ triggers survival pathways (60), and the binding of p62 (also known as sequestosome-1, required for both the formation and autophagic degradation of polyubiquitin-containing bodies) to its targets (61). Indeed, the regulation of NF-κB by the atypical kinase is relevant to Ras-induced oncogenesis (24, 30). These findings indicate that PKCζ is involved in the IKK signaling complex and, thus, in NF-κB activation.

Activated-NF-κB promotes cytokine production, including that of the positive growth-regulator IL-6, favoring chemoresistance. The importance of IL-6 signaling in mediating tumorigenesis has been examined in a number of studies, and in *in vivo* studies, IL-6 signaling promotes the growth of tumors (62). Within the tumor microenvironment, IL-6 binds to gp80/gp130, leading to Janus kinase (JAK) activation and phosphorylation of Stat3, which regulates the expression of genes that mediate cellular proliferation and prevent apoptosis (Figure 2) (63). PKCζ can control the production of IL-6. Loss of the kinase *in vivo* leads to increased tumorigenicity linked to the overproduction of IL-6 (26), which is sustained by an inflammatory condition characterized by an M1-type immunological response (64, 65). IL-6 is a known positive regulator of growth in human tumors, including liver and lung tumors (66); however, its production requires NF-κB and PKCζ (58, 67). IL-1 is known to induce the production of inflammatory cytokines, such as IL-6, through a transcriptional mechanism dependent on NF-κB activation (68, 69). Finally, PKCζ may regulate IL-6 promoter activity and transcription through C/EBPβ regulation via an NF-κB-independent mechanism (26). This finding suggests that PKCζ can both positively regulate NF-κB and, at the same time, regulate IL-6 transcription through independent pathways.

One pathway through which NF-κB can be activated is the Toll-like receptor (TLR) pathway, which occurs through the adapter protein myeloid differentiation primary response gene 88 (MyD88). NF-κB activation is a result of underlying inflammation or a consequence of the formation of an inflammatory microenvironment during malignant progression characterized by up-regulation of the tumor promoting cytokines IL-6 and TNF-α (70). Activation of the TNF receptor promotes NF-κB activation in breast cancer cells, leading to increased cancer cell survival and resistance to ionizing radiation (71). Elevated levels of activated-NF-κB induce cyclin D gene transcription and cell cycle progression, activation of anti-apoptotic genes *bcl-2* and *bcl-x_L_*, expression of vascular endothelial growth factor and consequent tumor angiogenesis, activation of transcription factor c-myc, metalloproteinase gene expression, and remodeling of the extra-cellular matrix (72–74).

Cancer-associated p53 mutants acquire significant pro-inflammatory activity mediated by NF-κB, which promotes both tumor initiation and tumor progression (75). Mutant p53 isoforms exhibit a distinct gain-of-function activity, enforcing a chronic state of TNF-α-induced NF-κB activation and resulting in persistent tissue damage, increased genomic instability, extended inflammation, and an augmented capacity for mutant p53-containing cells to evade apoptosis.

Altogether, these data confirm the involvement of the inflammatory tumor microenvironment in cancer, thus, attesting to the contribution of NF-κB activation in chemoresistance.

Recently, Levy and co-workers described a precise mechanism through which NF-κB activation is controlled directly by Rel A (a subunit of NF-κB) via the methyltransferase SETD6-mediated methylation of Lys310 (76). The methylated form of RelA recruits the G9a-related methyltransferase GLP and induces histone methylation, which represses the chromatin state of NF-κB-dependent genes, ensuring that they are not transcribed (Figure 2) (76). This event is coordinated by the PKC-ζ-dependent phosphorylation of Rel A on Ser31, leading to the release of GLP and the recruitment of CBP to RelA, followed by the acetylation of Lys310 and histones, resulting in enhanced transcription (Figure 2) (67). This mechanism for the RelA control of NF-κB in inflammation has been observed in PKCζ-null cells, which are incapable of mounting an efficient inflammatory response to TNF and IL-1. The phenotype of the immune system of PKCζ-null mice further supports the role of PKCζ in controlling NF-κB *in vivo* (58). These mice display alterations in the development of secondary lymphoid organs, showing morphological defects in the spleen and a reduction in the number of mature B cells (77). Furthermore, they exhibit defects in T helper 2 differentiation, IL-4 production, the nuclear translocation of Stat6 and Rel A (78) and liver damage, which was due to the depletion of protective signals in this organ (79).

## Conclusion

The links between inflammation and cancer have been the subject of recent studies, as the identification of the underlying molecular mechanisms may be highly relevant for cancer therapy. The first link between inflammation and cancer was suggested based on the use of anti-inflammatory therapies that have shown efficacy in cancer prevention and treatment (80).

The data described herein indicate that PKCζ is critical in the generation of inflammatory cytokines that might decide the final outcome of the neoplastic process. PKCζ exhibits both pro-inflammatory and anti-inflammatory effects, which complicates the interpretation of the findings published thus far. However, data from a study examining the mouse PKCζ-KO phenotype confirmed the critical contribution of this kinase to inflammation and cancer induction. PKCζ could be considered a tumor suppressor, though other studies have elucidated functional contributions of the connection of PKCζ to NF-κB and Stat3/IL-6 in carcinogenesis. The molecular mechanism by which PKCζ participates in multi-level regulation is strictly dependent on cell type and intracellular localization.

The data obtained from PKCζ-null mice describe the real biological contribution of the kinase, at least in part. Genetic ablation of PKCζ leads to a global, dramatic shutdown of the regulation of the master regulator proteins associated with inflammation, cancer, and apoptosis. Here, a limiting factor is that the available data do not permit the discrimination of individual molecular mechanisms in which PKCζ is involved.

An emerging concept is that the different functionalities of PKCζ are related to its intracellular distribution. The nucleus appears to be a functional site for PKCζ; this localization is regulated by oxidative stress, which is a condition present during both chemoresistance and inflammation. Nuclear-PKCζ redistribution reduces the sensitivity of cancer cells to chemotherapeutic agents, tagging this kinase as a useful target for tumor cell chemosensitization. Understanding the real molecular roles of nuclear PKCζ will be the next step in defining the specific mechanism that links oxidative stress, inflammation, and chemoresistance.

## Conflict of Interest Statement

The authors declare that the research was conducted in the absence of any commercial or financial relationships that could be construed as a potential conflict of interest.

## Abbreviations

AOM, azoxymethane; GLP, histone methyltransferase; IκBα, nuclear factor of kappa light polypeptide gene enhancer in B-cells inhibitor alpha; IKK, IKB kinase; IL-1, interleukin 1; IL-6, interleukin 6; LPS, lipopolysaccharide; MEFs, mouse embryonic fibroblasts; MEK5, mitogen-activated protein kinase 5; NES nuclear export signal; NF-κB, nuclear factor kappa B; NLS, nuclear localization signal; PAR-6, par6 cell polarity protein; PHGDH, 3-phosphoglycerate dehydrogenase; PI3K, phosphatidylinositol 3-kinase; PIs, phosphatidylinositols; PKC, protein kinase C; PS, pseudosubstrate; SETD6, SET domain containing 6; Stat3, signal transducer and activator of transcription 3; TNFα, tumor necrosis factor α.
